# Real-world applicability and impact of early rhythm control for European patients with atrial fibrillation: a report from the ESC-EHRA EORP-AF Long-Term General Registry

**DOI:** 10.1007/s00392-021-01914-y

**Published:** 2021-08-27

**Authors:** Marco Proietti, Marco Vitolo, Stephanie L. Harrison, Deirdre A. Lane, Laurent Fauchier, Francisco Marin, Michael Nabauer, Tatjana S. Potpara, Gheorghe-Andrei Dan, Giuseppe Boriani, Gregory Y. H. Lip, the ESC-EHRA EORP-AF Long-Term General Registry Investigators

**Affiliations:** 1 Liverpool Centre for Cardiovascular Science, University of Liverpool and Liverpool Heart and Chest Hospital, Liverpool, UK; 2 Department of Clinical Sciences and Community Health, University of Milan, Milan, Italy; 3 Geriatric Unit, IRCCS Istituti Clinici Scientifici Maugeri, Milan, Italy; 4 Cardiology Division, Department of Biomedical, Metabolic and Neural Sciences, Policlinico di Modena, University of Modena and Reggio Emilia, Modena, Italy; 5 Department of Clinical Medicine, Aalborg University, Aalborg, Denmark; 6 Service de Cardiologie, Centre Hospitalier Universitaire Trousseau, Tours, France; 7 Department of Cardiology, Hospital Universitario Virgen de la Arrixaca, IMIB-Arrixaca, University of Murcia, CIBERCV, Murcia, Spain; 8 Department of Cardiology, Ludwig-Maximilians-University, Munich, Germany; 9 School of Medicine, University of Belgrade, Belgrade, Serbia; 10 Intensive Arrhythmia Care, Cardiology Clinic, Clinical Center of Serbia, Belgrade, Serbia; 11 Colentina University Hospital, ‘Carol Davila’ University of Medicine, Bucharest, Romania

**Keywords:** Atrial fibrillation, Rhythm control, Rate control, Outcomes

## Abstract

**Background:**

Use of rate/rhythm control is essential to control symptoms in patients with atrial fibrillation (AF). Recently, the EAST-AFNET 4 trial described how early rhythm control strategy was associated with a lower risk of adverse clinical outcomes.

**Objectives:**

The aim was to evaluate the real-world applicability and impact of an early rhythm control strategy in patients with AF.

**Methods:**

Use of an early rhythm control strategy was assessed in a European cohort of AF patients derived from the EHRA-ESC EORP-AF General Long-Term Registry. Early rhythm control was defined as use of antiarrhythmic drugs or cardioversion/catheter ablation. The primary outcome included cardiovascular death, stroke, acute coronary syndrome, and worsening of heart failure. Quality of life and health-care resource usage were also assessed as outcomes.

**Results:**

Among the 10,707 patients evaluated for eligibility to EAST-AFNET 4, a total of 3774 (34.0%) were included. Early rhythm control was associated with better quality of life, but with greater use of health-care resources. During follow-up, the primary outcome occurred less often in early rhythm control patients than in those with no rhythm control (13.6% vs. 18.5%, *p* < 0.001). In the multivariate adjusted Cox regression model, no significant difference was found between no rhythm control and early rhythm control, for the primary outcome. No difference in the primary outcome between early rhythm control and ‘no rhythm control patients’ adherent to Atrial fibrillation Better Care (ABC) pathway’ was evident (*p* = 0.753)

**Conclusions:**

Use of an early rhythm control strategy was associated with a lower rate of major adverse events, but this difference was non-significant on multivariate analysis, being mediated by differences in baseline characteristics and clinical risk profile. Early rhythm control was associated with a higher use of health-care resources and risk of hospital admission, despite showing better quality of life.

**Graphic abstract:**

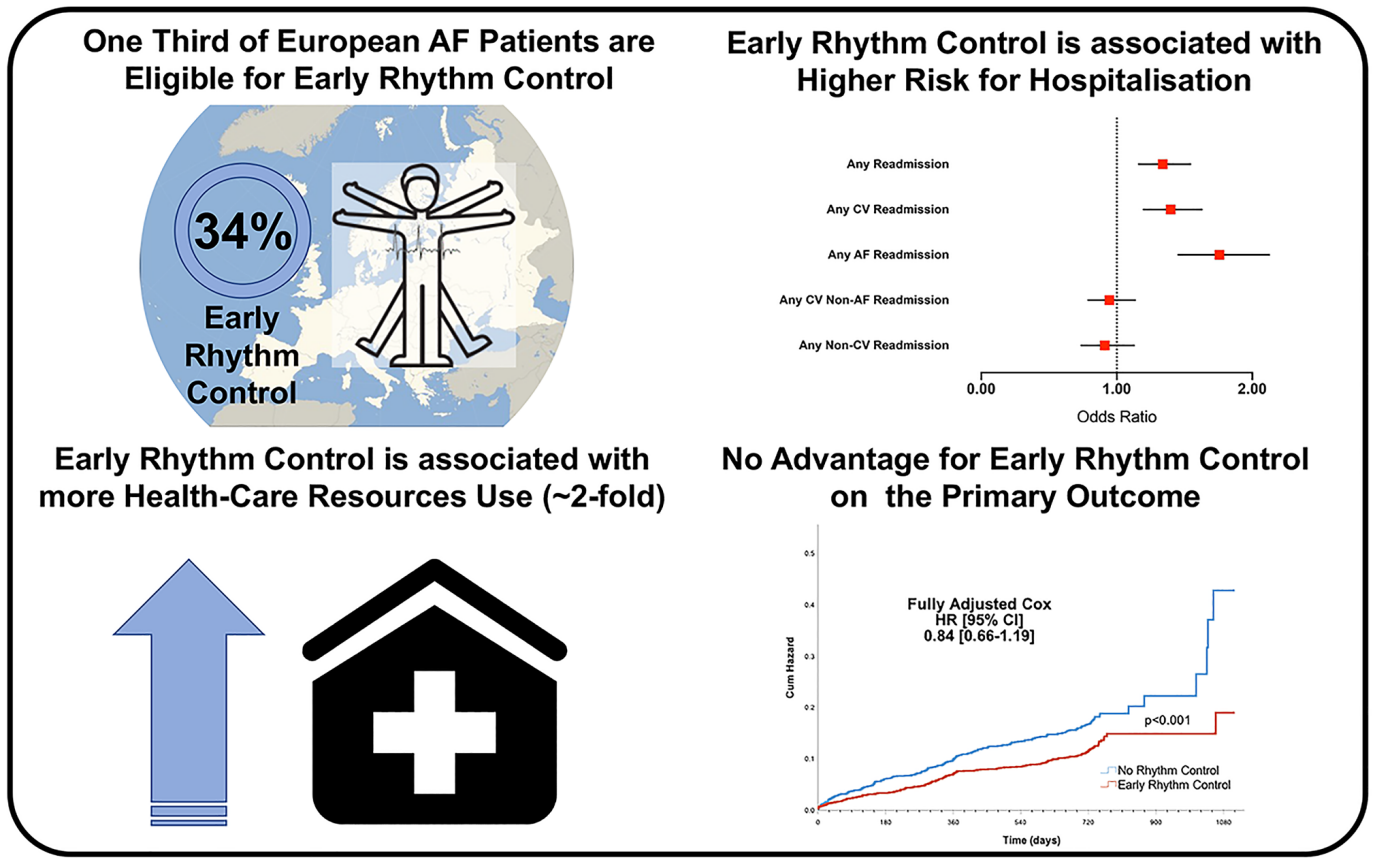

**Supplementary Information:**

The online version contains supplementary material available at 10.1007/s00392-021-01914-y.

## Introduction

Patient-centred, symptom-directed decisions on rate or rhythm control are pivotal considerations for the clinical management of patients with atrial fibrillation (AF). This approach is recommended by the European Society of Cardiology (ESC) 2020 Clinical Guidelines for AF management [1] and is part of the ‘B’ criterion of the Atrial fibrillation Better Care (ABC) pathway for the integrated care management of patients with AF [2].

It has long been debated whether rate vs. rhythm control strategy could have differential impact on major clinical outcomes, especially the risk of all-cause death occurrence [3–5]. A more recent approach advocated is that a background rate control therapy is needed in all AF patients, with additional rhythm control strategies to mitigate symptom burden [1, 6].

The “Early Treatment of Atrial Fibrillation for Stroke Prevention Trial” (EAST-AFNET 4) study [7] tested the hypothesis that early initiation of rhythm control therapy conferred an advantage in terms of the risk of major adverse events. Over a median 5.1 years of follow-up, the trial reported a 21% relative risk reduction (6–34% CI) for a composite primary outcome of cardiovascular (CV) death, stroke, acute coronary syndrome and hospitalization/worsening of heart failure [7]. Given that the intervention arm had structured follow-up and a ‘package of care’, the applicability of this trial to real world clinical practice requires further study.

In this ancillary analysis from the ESC-European Heart Rhythm Association (EHRA) EURObservational Research Programme (EORP) AF General Long-Term Registry, the aim of this study was to utilize a large real-world cohort of European AF patients: first, to determine how many patients would be able to fulfil the EAST-AFNET 4 inclusion criteria; and second, to test the impact of an early rhythm control strategy on the use of health care resources, quality of life and major adverse events.

## Methods

The ESC-EHRA EORP AF General Long-Term Registry is a multicentre observational registry held by the ESC and endorsed by the EHRA, with the General Long-Term Registry preceded by the EORP-AF General Pilot Registry [8–11]. The EORP-AF General Long-Term Registry is a prospective, observational, multicentre registry established by ESC in 27 participating countries. The study enrolled consecutive patients with AF presenting in 250 cardiology practices, in both in- and outpatient settings. The detailed description of the study design, baseline characteristics and 1-year follow-up results have been provided previously [12, 13]. Briefly, all patients enrolled had AF documented within 12 months before enrolment on the basis of objective electrocardiographic evaluation. All patients were aged ≥ 18 years and provided written informed consent. Enrolment was undertaken from October 2013 to September 2016, with 1-year and 2-year follow-up. Institutional review board approved the study protocol for each country, and the study was performed according to the EU Note for Guidance on Good Clinical Practice CPMP/ECH/135/95 and the Declaration of Helsinki.

Symptomatic status was defined according to EHRA score [1]. Thromboembolic risk was defined according to the CHA_2_DS_2_-VASc score [14], with bleeding risk defined by the HAS-BLED score [15]. Multimorbidity was defined as the concomitant presence of at least two different long-term comorbidities. Frailty was defined on the basis of a 38-items frailty index of ≥ 0.25, according to Rockwood and Mitnitski [16]. Polypharmacy was classified as the concomitant prescription of ≥ 5 drugs [17]. Adherence of clinical management to the ‘Atrial fibrillation Better Care’ (ABC) pathway was retrospectively defined in this cohort, as previously reported [18]. Briefly, the ABC pathway has been proposed to streamline integrated care and holistic management in AF patients and is based on the following: (i) avoid stroke with anticoagulation; (ii) better symptom management with patient-centred symptom-directed decisions on rate or rhythm control; (iii) cardiovascular risk factor and comorbidities optimization including lifestyle changes [2]. Adherence to the ABC pathway has consistently been associated with reduction in risk for major clinical outcomes associated with AF [19].

### Evaluation of eligibility to EAST-AFNET 4 trial criteria

To understand how many patients in the current cohort would have been eligible to be enrolled in the EAST-AFNET 4, we applied the same inclusion criteria to our population [20]. All the patients included in the EORP-AF General Long-Term Registry were ≥ 18 years and diagnosed with AF within 12 months of enrolment. Hence, we included all the patients that were aged ≥ 75 years or had a clinical history positive for previous stroke or transient ischaemic attack. Additionally, we included all the patients that fulfilled at least two of the following criteria: (i) age ≥ 65 years; (ii) female sex; (iii) hypertension; (iv) diabetes mellitus; (v) previous myocardial infarction or any coronary revascularization procedure; (vi) stable heart failure (NYHA II or ejection fraction < 50%); (vii) left ventricular hypertrophy: (viii) chronic kidney disease (creatinine clearance 15–59 mL/min); (ix) peripheral arterial disease.

### Definition of early rhythm control

Early rhythm control was defined at the moment of enrolment visit or discharge following hospital admission. All those patients who received a rhythm control intervention during the index episode, such as electrical cardioversion, pharmacological cardioversion, catheter ablation, or were prescribed an antiarrhythmic drug (Class Ia, Class Ic, Class III) at discharge, were included in the ‘early rhythm control’ group.

All the other patients prescribed beta blockers, digoxin, or non-dihydropyridine calcium-channel blockers, as rate control drugs, were included in the ‘no rhythm control’ group.

### Evaluation of quality of life

Quality of life was evaluated at baseline and 1-year follow-up using the EQ-5D-5L questionnaire, a generic, extensively validated, easy to use instrument that consists of two parts: the EQ-5D descriptive system and the EQ visual analogue scale (https://euroqol.org/eq-5d-instruments/eq-5d-5l-about/). The descriptive system consists of five dimensions (mobility, self-care, usual activities, pain/discomfort and anxiety/depression) with five possible levels for each dimension (no problems, slight problems, moderate problems, severe problems and extreme problems), generating 5^5^ = 3125 unique health states. According to a previous report, using the UK trade-off value set we translated each of the levels into a single numeric value, with the lowest values corresponding to better health [21]. Furthermore, combining the single values, we translated the five-digit health state into a single index, the Health Utility Score (HUS) by subtracting each value from 1. The best possible health in each dimension (= 11,111) corresponded to an HUS of 1.0 (perfect health). An HUS of 0 is equivalent to death. The visual analogue scale was used for patients to self-rate their current health status, ranging from 0 (worst health imaginable) to 100 (best health imaginable).

### Evaluation of health-care resources use

We examined the differential use of health-care resources according to the use of early rhythm control. In patients enrolled during hospitalization, we evaluated the overall length of stay. Further, we analysed the occurrence and number of cardiology and internal medicine/general practitioner visits, as well as the emergency room (ER) admissions during the follow-up observation (at 1 and 2 years of follow-up).

Also, to account comprehensively for the use of health-care resources, we evaluated the occurrence of hospital admission/readmission throughout follow-up observation, as follows: (i) any readmission; (ii) any CV readmission; (iii) any AF readmission; (iv) any CV non-AF readmission; and (v) any non-CV readmission.

### Major adverse events

The primary main outcome of the study was equivalent to the original EAST-AFNET 4 trial primary outcome, being a composite of (i) CV death; (ii) any stroke; (iii) worsening heart failure; and (iv) acute coronary syndrome. The secondary clinical outcomes were: (i) major adverse cardiovascular events (MACEs): as the composite of any thromboembolic events, any acute coronary syndrome and CV death; and (ii) all-cause death.

### Statistical analysis

Continuous variables were expressed as mean SD or median IQR and differences across the groups were evaluated according to Student’s *T* test and Mann–Whitney *U* test, respectively. Categorical variables were expressed as counts and percentages and differences across groups were evaluated according to the Chi-square test. A univariate and multivariate logistic regression model was compiled to evaluate the clinical factors associated with the choice of early rhythm control.

To evaluate the relationship between the use of early rhythm control and length of hospital stay, in addition to the number of medical visits during the follow-up, we performed a linear regression analysis adjusted for type of AF, CHA_2_DS_2_-VASc score and EHRA score. To analyse the association between the use of early rhythm control and the occurrence of medical visits, a logistic regression model was used, adjusted for type of AF, CHA_2_DS_2_-VASc score and EHRA score. We also performed a linear regression analysis adjusted for type of AF, CHA_2_DS_2_-VASc score and EHRA score to evaluate the relationship between the use of early rhythm control and quality of life measures at baseline and throughout follow-up. The association between the use of early rhythm control and readmission outcomes was tested using a logistic regression model, adjusted for type of AF, CHA_2_DS_2_-VASc score, EHRA score and use of OAC.

Differences in cumulative risk for the main study outcome were evaluated using log-rank tests and Kaplan–Meier curves. To investigate the independent associations between the use of early rhythm control and the primary and main secondary clinical outcomes, a Cox regression analysis was performed. Two different models were constructed: (i) adjusted for type of AF, CHA_2_DS_2_-VASc score and EHRA score and use of oral anticoagulant (OAC); (ii) adjusted for type of AF, EHRA score, age, sex, hypertension, diabetes mellitus, heart failure, severe coronary artery disease, valvular disease, left ventricular hypertrophy, peripheral artery disease, stroke/transient ischaemic attack, chronic kidney disease, chronic obstructive pulmonary disease, malignancy and use of OAC. Lastly, we performed a sensitivity analysis regarding the occurrence of study primary outcome, comparing the early rhythm control strategy to a ‘no rhythm control’ approach adherent to the ABC pathway.

All linear regression analyses were reported as Beta coefficient and 95% CI. All logistic regression analyses results were reported as OR and 95% CI. All Cox regression analyses results were reported as HR and 95% CI. A two-sided *p* < 0.05 was considered statistically significant. All analyses were performed using SPSS statistical software version 25.0.0.1 (IBM, NY, USA) for MacOS.

## Results

From the original 11,096 patients enrolled in the EORP-AF, 10,707 (96.5%) were evaluated for eligibility to EAST-AFNET 4 inclusion criteria, due to missing data for the variables use to evaluate eligibility. Overall, 3774 (34.0%) patients fulfilled the study’s inclusion criteria and were selected for this analysis. Among those not selected, 2654 (38.3%) did not fulfil the study main criteria; 3682 (53.1%) did not fulfil the study additional criteria; 4013 (57.9%) had long-standing persistent or permanent AF or did not report the type of AF.

Comparing EORP-AF patients included in this analysis to those not included, included patients were older, with higher CHA_2_DS_2_-VASc and HAS-BLED scores and with more comorbidities and prescribed medications (all *p* < 0.001). EORP-AF patients qualifying for inclusion had a higher proportion of those aged ≥ 75 years, were more likely to have persistent AF and had a higher symptom burden compared to those enrolled in the EAST-AFNET 4 study (Table 1). Also, EAST-AFNET 4 patients reported higher proportions of hypertension and diabetes mellitus, while EORP-AF patients had more structural heart disease (heart failure, severe coronary artery disease, valvular disease, left ventricular hypertrophy) (Table 1).

**Table 1:** Baseline characteristics in EAST-AFNET 4 and EORP-AF and according to early rhythm control prescription

Variables	EAST-AFNET 4 (*N* = 2789)	EORP-AF (*N* = 3774)	No rhythm control (*N* = 1722)	Early rhythm control (*N* = 2052)	p*
Age, years mean (SD)	70.3 (8.3)	69.8 (10.4)	71.9 (10.3)	68.0 (10.2)	< 0.001
Age, years median [IQR]	71 [66–76]	71 [63–78]	74 [66–79]	69 [62–76]	< 0.001
Age ≥ 75 years, *n* (%)	812 (29.1)	1483 (39.3)	855 (49.7)	628 (30.6)	< 0.001
Male sex, *n* (%)	1496 (53.6)	2026 (53.7)	878 (51.0)	1148 (55.9)	0.002
BMI, kg/m^2^ median [IQR]	28.6 [25.5–32.1]	27.8 [24.8–31.2]	27.5 [24.6–31.2]	27.9 [25.0–31.3]	0.024
Type of AF, *n* (%)					< 0.001
First detected	1048 (37.6)	1031 (27.3)	564 (32.8)	467 (22.8)	
Paroxysmal	994 (35.7)	1538 (40.8)	742 (43.1)	796 (38.8)	
Persistent	743 (26.7)	1205 (31.9)	416 (24.2)	789 (38.5)	
EHRA score, *n* (%)					< 0.001
EHRA I	801 (30.4)	1390 (36.8)	784 (45.5)	606 (29.5)	
EHRA II	1358 (51.6)	1465 (38.8)	581 (33.7)	884 (43.1)	
EHRA III	447 (17.0)	808 (21.4)	310 (18.0)	498 (24.3)	
EHRA IV	27 (1.0)	110 (2.9)	47 (2.7)	63 (3.1)	
Hypertension, *n* (%)	2450 (87.8)	2559 (68.0)	1122 (65.4)	1437 (70.1)	0.002
Diabetes mellitus, *n* (%)	694 (24.9)	860 (22.8)	440 (25.6)	420 (20.5)	< 0.001
NYHA class, *n* (%)					< 0.001
No HF	1819 (65.3)	2360 (62.5)	1019 (59.2)	1341 (65.4)	
I	331 (11.9)	239 (6.3)	125 (7.3)	114 (5.6)	
II	514 (18.5)	712 (18.9)	325 (18.9)	387 (18.9)	
III	120 (4.3)	396 (10.5)	214 (12.4)	182 (8.9)	
IV	–	67 (1.8)	39 (2.3)	28 (1.4)	
Prior stroke/TIA, *n* (%)	328 (11.8)	379 (10.1)	191 (11.2)	188 (9.2)	0.049
Severe CAD, *n* (%)	479 (17.2)	827 (21.9)	392 (22.8)	435 (21.2)	0.247
Valvular disease, *n* (%)	1251 (45.0)	1807 (48.6)	847 (50.3)	960 (47.2)	0.064
LVH, *n* (%)	132 (4.7)	988 (28.5)	463 (30.1)	525 (27.3)	0.070
PAD, *n* (%)	122 (4.4)	303 (8.1)	142 (8.4)	161 (7.9)	0.608
CKD, *n* (%)	351 (12.6)	454 (12.0)	234 (13.6)	220 (10.7)	0.006
COPD, *n* (%)	209 (7.5)	279 (7.4)	168 (9.8)	111 (5.4)	< 0.001
Malignancy, *n* (%)					0.003
No malignancy	2563 (92.2)	3467 (91.9)	1560 (90.6)	1907 (92.9)	
Active malignancy	19 (0.7)	79 (2.1)	52 (3.0)	27 (1.3)	
Prior malignancy	197 (7.1)	216 (5.7)	104 (6.0)	112 (5.5)	
CHA_2_DS_2_-VASc, mean (SD)	3.3 (1.3)	3.3 (1.6)	3.6 (1.6)	3.1 (1.5)	< 0.001
CHA_2_DS_2_-VASc, median [IQR]	3 [2–4]	3 [2–4]	4 [2–5]	3 [2–4]	< 0.001
HAS-BLED, mean (SD)	–	1.6 (1.0)	1.7 (1.1)	1.5 (1.0)	< 0.001
HAS-BLED, median [IQR]	–	2 [1, 2]	2 [1, 2]	1 [1, 2]	< 0.001
Multimorbidity, *n* (%)	–	2681 (83.3)	1218 (84.3)	1463 (82.4)	0.158
Frailty, *n* (%)	–	555 (21.7)	236 (20.6)	319 (22.5)	0.246
Polypharmacy, *n* (%)	–	2375 (63.3)	999 (58.3)	1376 (67.5)	< 0.001
ABC pathway adherence, *n* (%)	–	758 (29.9)	344 (29.3)	414 (30.5)	0.483
ABC pathway criteria, *n* (%)	–				0.335
0		59 (2.3)	33 (2.8)	26 (1.9)	
1		494 (19.5)	239 (20.3)	255 (18.8)	
2		1221 (48.2)	560 (47.6)	661 (48.7)	
3		758 (29.9)	344 (29.3)	414 (30.5)	
Pharmacological treatments					
Any antiplatelet, *n* (%)	455 (16.4)	921 (24.4)	441 (25.6)	480 (23.4)	0.111
Any OAC, *n* (%)	2517 (90.5)	3255 (86.3)	1426 (82.8)	1829 (89.2)	< 0.001
Any VKA, *n* (%)	–	1644 (43.6)	687 (39.9)	957 (46.7)	< 0.001
Any NOAC, *n* (%)	–	1613 (42.8)	740 (43.0)	873 (42.6)	0.777
ACEi/ARBs, *n* (%)	1932 (69.4)	2541 (67.4)	1156 (67.3)	1385 (67.5)	0.875
Diuretics, *n* (%)	1120 (40.3)	1900 (50.4)	902 (52.5)	998 (48.7)	0.020
MRAs, *n* (%)	182 (6.5)	577 (15.3)	276 (16.1)	301 (14.7)	0.240
Statins, *n* (%)	1196 (43.0)	1832 (48.6)	843 (49.0)	989 (48.3)	0.636
Oral antidiabetics, *n* (%)	459 (16.5)	599 (15.9)	314 (18.3)	285 (13.9)	< 0.001
Insulin, *n* (%)	121 (4.3)	205 (5.4)	102 (5.9)	103 (5.0)	0.220

*ABC* atrial fibrillation better care, *AF* atrial fibrillation, *BMI* body mass index, *CAD* coronary artery disease, *CKD* chronic kidney disease, *COPD* chronic obstructive pulmonary disease, *EHRA* European Heart Rhythm Association, *HF* heart failure, *IQR* interquartile range, *LVH* left ventricular hypertrophy, *NYHA* New York Heart Association, *PAD* peripheral artery disease, *SD* standard deviation, *TIA* transient ischaemic attack

*p value is referred to the comparison between no rhythm control and early rhythm control

### Determinants of early rhythm control

Among EORP-AF patients, 2052 (54.4%) were treated according to an early rhythm control strategy and 1722 (45.6%) with no rhythm control at baseline. Patients prescribed early rhythm control were younger, more likely male, with persistent AF and a higher burden of AF symptoms. The early rhythm control group had more prevalent hypertension, but less comorbidities, with lower thromboembolic and bleeding risk factors, and a greater proportion receiving OAC (Table 1). Mean (SD) time from AF diagnosis to enrolment was not significantly different between patients treated with and without early rhythm control [48.6 (84.4) days vs. 51.2 (88.6) days, respectively; *p* = 0.426], nor was the mean (SD) time from enrolment to discharge [0.4 (14.1) days vs. 1.5 (16.7) days, respectively; *p* = 0.381].

**Fig. 1:**
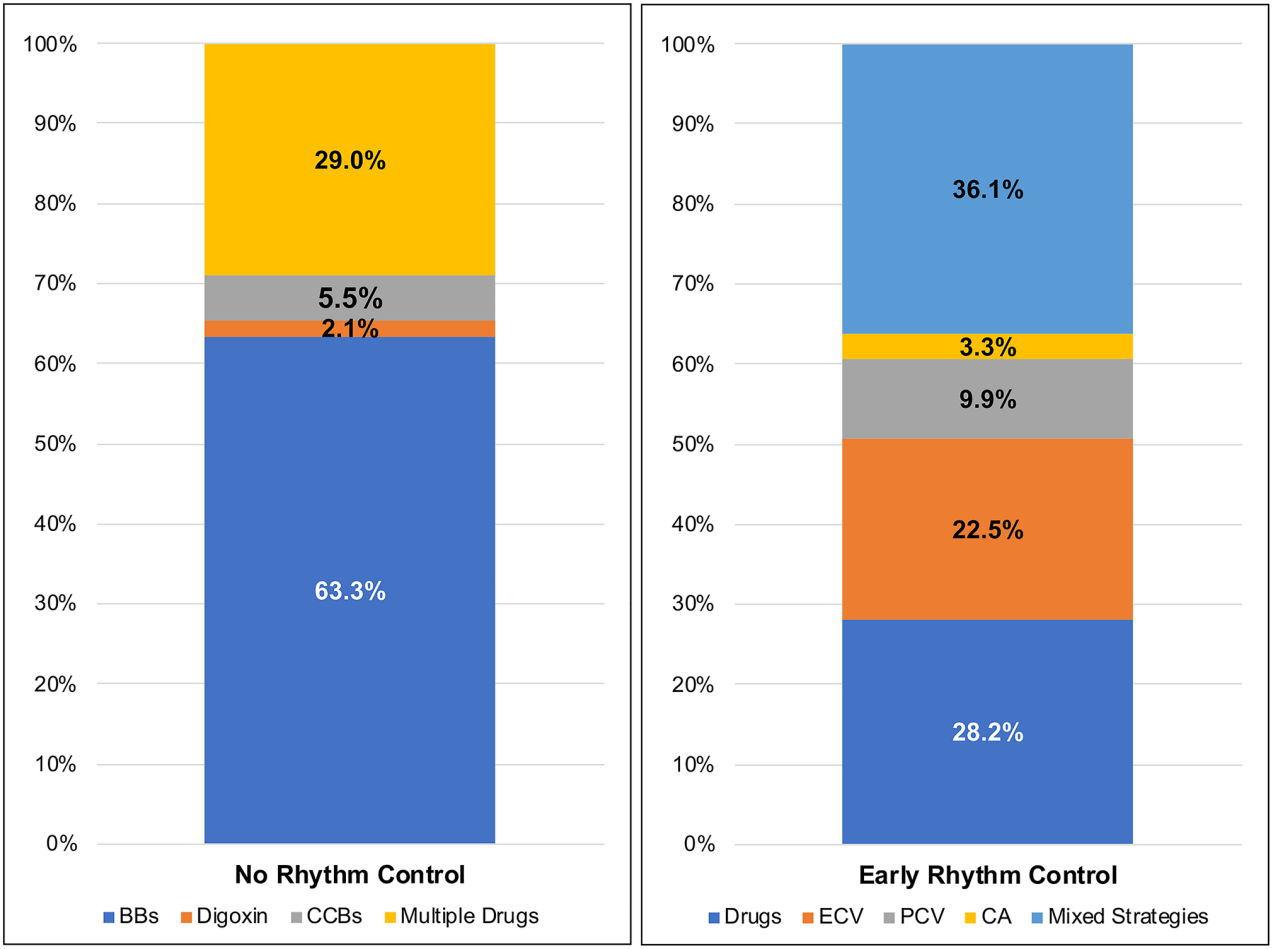
Distribution of rate/rhythm control treatments according to groups at baseline. *BBs* beta blockers, *CA* catheter ablation, *CCBs* calcium-channel blockers, *ECV* electric cardioversion, *PCV* pharmacological cardioversion

At discharge, among patients not prescribed early rhythm control, a rate control strategy based on the use of beta blockers was most commonly used (63.3%), while 29.0% were managed with a combination of rate control drugs and only a minority were treated with digoxin only (2.1%) and non-dihydropyridine calcium-channel blockers (5.5%). (Fig. 1). In the early rhythm control group, use of a combination antiarrhythmic strategy (antiarrhythmic drugs + cardioversion/ablation) was most common (36.1%), with sole use of antiarrhythmic drugs only in 28.2% (Fig. 1). Electric cardioversion only was used in 22.5% of patients, pharmacological cardioversion only in 9.9% and catheter ablation only in 3.3% (Fig. 1).

Based on the differences in baseline characteristics, univariate logistic regression analysis for the clinical factors associated with the use of an early rhythm control strategy, followed by a multivariate model (Table S1), showed that paroxysmal and persistent AF, progressively increasing symptom burden and presence of hypertension were significantly associated with the use of an early rhythm control strategy. Increasing age, higher NYHA class and concomitant diabetes mellitus, stroke/transient ischaemic attack and chronic obstructive pulmonary disease were all inversely associated with early rhythm control (Table S1).

### Evaluation of quality of life

At baseline, evaluation of quality of life showed that patients managed with early rhythm control were more able to attend to their self-care (*p* = 0.001) and their usual activities (*p* < 0.001), but were more likely anxious, even after adjustment for CHA_2_DS_2_-VASc score, type of AF and EHRA score (Table 2). In the multivariate analysis, at 1-year follow-up, those patients with early rhythm control had better levels of mobility (*p* < 0.001), self-care (*p* = 0.009) and participation in their usual activities (*p* = 0.005), with an overall better health state according to the HUS (*p* = 0.002), compared to those not prescribed with an early rhythm control. At 2 years follow-up, patients prescribed early rhythm control reported less impairment in all five dimensions of the EQ-5D-5L questionnaire and a better overall health state according to both HUS (*p* < 0.001) and VAS (*p* = 0.053), after adjustment for CHA_2_DS_2_-VASc score, type of AF and EHRA score (Table 2).

**Table 2:** Quality of life indicators according to early rhythm control

	No rhythm control	Early rhythm control	*p*	Early rhythm control	*p*
				Beta (95% CI)*	
Baseline					
EQ-5D-5L mobility	0.049 (0.063)	0.043 (0.061)	0.019	− 0.003 (− 0.007/0.001)	0.156
EQ-5D-5L self-care	0.020 (0.039)	0.014 (0.035)	< 0.001	− **0.005 (0.007/** − **0.002)**	**0.001**
EQ-5D-5L usual activities	0.037 (0.047)	0.031 (0.044)	< 0.001	− **0.006 (**− **0.009/** − **0.003)**	< **0.001**
EQ-5D-5L pain/discomfort	0.049 (0.068)	0.044 (0.063)	0.024	− 0.004 (− 0.009/0.000)	0.062
EQ-5D-5L anxiety	0.046 (0.066)	0.054 (0.072)	0.001	**0.007 (0.002/0.012)**	**0.004**
Health utility score	0.80 (0.21)	0.81 (0.19)	0.068	0.010 (− 0.004/0.025)	0.142
Visual analogue scale	68.3 (20.6)	68.3 (20.8)	0.992	0.093 (− 1.486/1.673)	0.908
1-year follow-up					
EQ-5D-5L mobility	0.046 (0.059)	0.036 (0.056)	< 0.001	− **0.009 (**− **0.014/** − **0.004)**	< **0.001**
EQ-5D-5L self-care	0.018 (0.035)	0.013 (0.033)	0.002	− **0.004 (**− **0.007/** − **0.001)**	**0.009**
EQ-5D-5L usual activities	0.033 (0.042)	0.027 (0.041)	0.001	− **0.005 (**− **0.009/** − **0.002)**	**0.005**
EQ-5D-5L pain/discomfort	0.044 (0.067)	0.039 (0.062)	0.056	− 0.005 (− 0.010/0.001)	0.118
EQ-5D-5L anxiety	0.042 (0.066)	0.040 (0.057)	0.536	− 0.004 (− 0.009/0.002)	0.197
Health utility score	0.817 (0.203)	0.844 (0.185)	0.001	**0.026 (0.009/0.043)**	**0.002**
Visual analog scale	69.7 (19.8)	70.9 (19.8)	0.169	1.283 (− 0.505/3.071)	0.159
2 years follow-up					
EQ-5D-5L mobility	0.051 (0.062)	0.040 (0.056)	< 0.001	− **0.009 (**− **0.015/** − **0.004)**	**0.001**
EQ-5D-5L self-care	0.021 (0.040)	0.012 (0.027)	< 0.001	− **0.009 (**− **0.012/** − **0.005)**	< **0.001**
EQ-5D-5L usual activities	0.038 (0.045)	0.028 (0.041)	< 0.001	− **0.008 (**− **0.012/** − **0.004)**	< **0.001**
EQ-5D-5L pain/discomfort	0.048 (0.068)	0.039 (0.062)	0.004	− **0.008 (**− **0.014/** − **0.001)**	**0.016**
EQ-5D-5L anxiety	0.043 (0.065)	0.037 (0.058)	0.052	− **0.007 (**− **0.013/** − **0.001)**	**0.020**
Health utility score	0.80 (0.21)	0.84 (0.18)	< 0.001	**0.042 (0.023/0.060)**	< **0.001**
Visual analogue scale	69.8 (19.4)	72.1 (19.8)	0.016	**1.943 (**− **0.026/3.911)**	**0.053**

Bold values depict significant association

*CI* confidence interval; for other acronyms please see previous tables’ legends

*Adjusted for CHA_2_DS_2_-VASc score, type of AF, EHRA score

### Evaluation of health-care resources use

Use of health-care resources is reported in Table 3. Patients managed with early rhythm control attended more cardiology and internal medicine/general practitioner appointments at both follow-up time points (all *p* < 0.001) (Table 3, upper panel).

**Table 3:** Use of health-care resources according to early rhythm control use

	No rhythm control	Early rhythm control	*p*	Early rhythm control	*p*
				OR (95% CI)*	
Cardiology visits 1Y, *n* (%)	940 (68.3)	1386 (83.0)	< 0.001	**2.07 (1.73–2.47)**	< **0.001**
IM/GP visits 1Y, *n* (%)	481 (43.9)	822 (58.9)	< 0.001	**1.87 (1.58–2.21)**	< **0.001**
ER admissions 1Y, *n* (%)	290 (21.6)	371 (22.4)	0.577	1.08 (0.90–1.29)	0.422
Cardiology visits 2Y, *n* (%)	738 (61.6)	1130 (76.2)	< 0.001	**1.88 (1.58–2.23)**	< **0.001**
IM/GP visits 2Y, *n* (%)	430 (42.1)	760 (58.9)	< 0.001	**2.02 (1.70–2.40)**	< **0.001**
ER admissions 2Y, *n* (%)	181 (15.8)	242 (16.8)	0.497	1.08 (0.87–1.35)	0.482

	No rhythm control	Early rhythm control	*p*	Early rhythm control	*p*
				Beta (95% CI)*	
Length of stay, days mean (SD)	7.6 (7.1)	5.9 (6.7)	< 0.001	**−** **0.992 (−** **1.572/** **−** **0.412)**	**0.001**
Cardiology visits 1Y, *N* mean (SD)	2.4 (1.9)	2.7 (2.2)	< 0.001	**0.349 (0.168/0.530)**	< **0.001**
IM/GP visits 1Y, *N* mean (SD)	3.9 (3.4)	4.6 (3.9)	0.004	**0.589 (0.115/1.062)**	**0.015**
ER admissions 1Y, *N* mean (SD)	1.7 (1.3)	1.7 (1.4)	0.704	0.005 (**−** 0.209/0.219)	0.963
Cardiology visits 2Y, *N* mean (SD)	2.0 (1.4)	2.2 (1.8)	0.004	**0.184 (0.025/0.342)**	**0.023**
IM/GP visits 2Y, *N* mean (SD)	3.5 (3.4)	3.7 (3.3)	0.505	0.094 (**−** 0.348/0.536)	0.676
ER admissions 2Y, *N* mean (SD)	1.6 (1.2)	1.6 (1.3)	0.610	0.061 (**−** 0.193/0.315)	0.637

	No rhythm control	Early rhythm control	*p*	Early rhythm control	*p*
				OR (95% CI)^†^	
Any readmission, *n* (%)	624 (38.6)	857 (46.2)	< 0.001	**1.34 (1.16–1.54)**	< **0.001**
Any CV readmission, *n* (%)	415 (25.7)	607 (32.7)	< 0.001	**1.40 (1.20–1.63)**	< **0.001**
Any AF readmission, *n* (%)	205 (12.7)	408 (22.0)	< 0.001	**1.76 (1.45–2.12)**	< **0.001**
Any CV non-AF readmission, *n* (%)	303 (18.8)	317 (17.1)	0.203	0.95 (0.79–1.14)	0.540
Any non-CV readmission, *n* (%)	207 (12.8)	213 (11.5)	0.232	0.91 (0.74–1.13)	0.369

Bold values depict significant association

*1Y* 1-year follow-up, *2YR* 2 years follow-up, *ER* emergency room, *GP* general practitioner, *IM* internal medicine, *OR* odds ratio; for other acronyms please see previous tables’ legends.

*Adjusted for CHA_2_DS_2_-VASc score, type of AF, EHRA score

^†^Adjusted for CHA_2_DS_2_-VASc score, type of AF, EHRA score, use of OAC

Among hospitalized patients, those prescribed early rhythm control had a shorter mean hospital stay, even after adjustment (*p* = 0.001) (Table 3, lower panel). Among those who attended cardiology visits, those with early rhythm control reported a higher number of visits both at 1 year (*p* < 0.001) and 2 years (*p* = 0.023) of follow-up. Among patients attending internal medicine/general practitioner visits, those with early rhythm control attended more often within the first year of follow-up than those with ‘no rhythm control’ (*p* = 0.015).

Patients treated with an early rhythm control strategy had a higher rate of any readmission, and admissions related to CV reasons or AF (all *p* < 0.001) (Table 3). Logistic regression, adjusted for type of AF, EHRA score, CHA_2_DS_2_-VASc score and use of OAC, found that an early rhythm control strategy was associated with higher odds of any hospital readmission, any CV readmission and any AF readmission (Table 3).

### Major adverse events

**Fig. 2:**
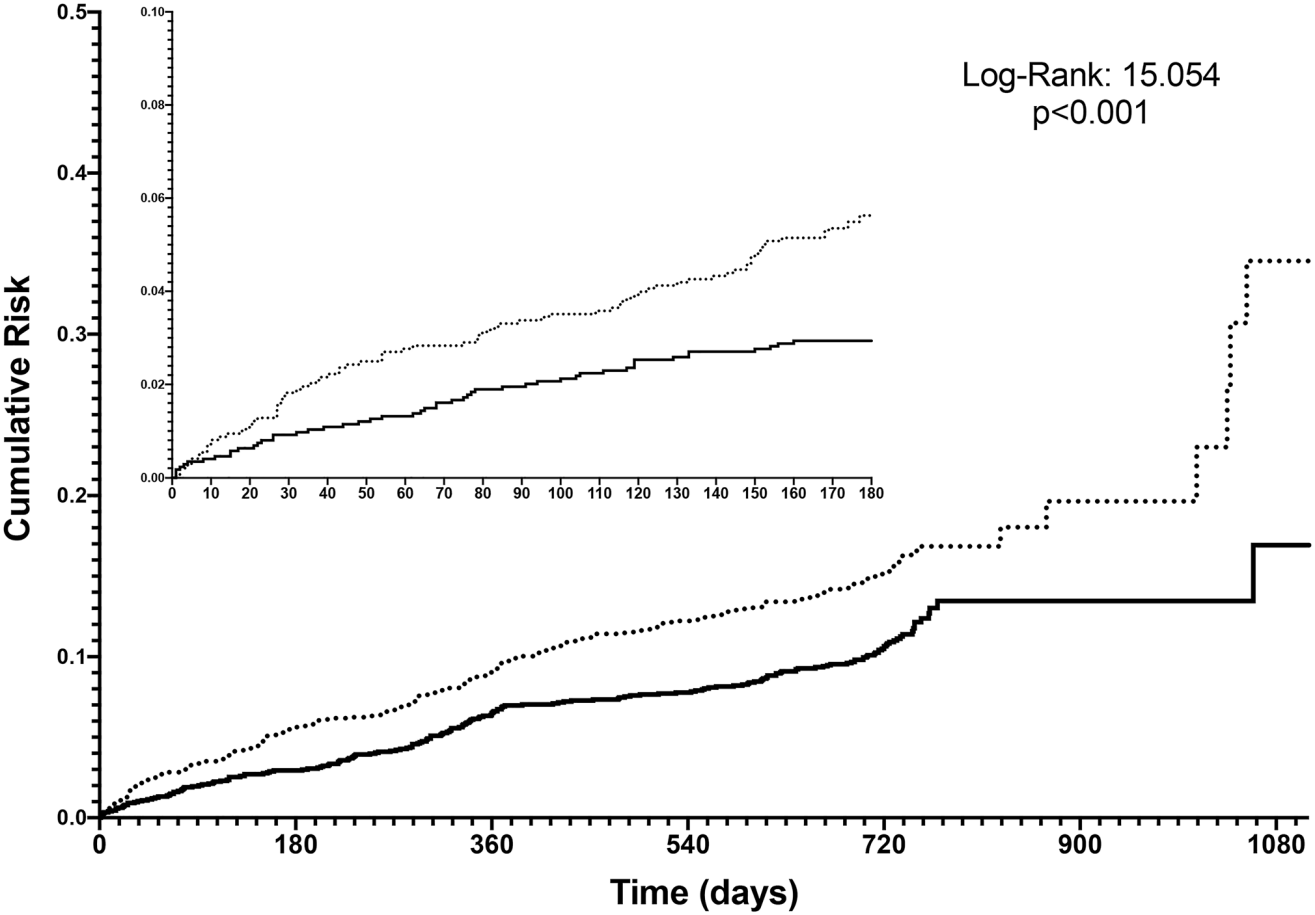
Kaplan–Meier curves for EAST-AFNET 4 primary outcome. Black solid line = early rhythm control; black dotted line = no early rhythm control

Follow-up data were available for 3354 (88.9%) of the patients. Over a mean (SD) follow-up of 675.4 (181.3) days, a total of 532 (14.1%) EAST-AFNET 4 defined primary outcome events were reported with an overall incidence of 8.9 per 100 patient-years. Death occurred in 321 (8.5%) and 380 (10.1%) experienced MACEs. Compared to those treated with an early rhythm control approach, the no rhythm control group had a higher proportion of patients with the EAST-AFNET 4 defined primary outcome, MACEs and all-cause death events (Table 4). The incidence of EAST-AFNET 4 defined primary outcome was 10.8 per 100 patient-years in patients with no rhythm control and 7.4 per 100 patient-years in patients with early rhythm control. Kaplan–Meier curves for the EAST-AFNET 4 trial defined primary outcome showed that an early rhythm control strategy was associated with a progressively lower cumulative risk (*p* < 0.001) (Fig. 2).

**Table 4:** Major clinical outcomes comparing early rhythm control versus no rhythm control in the EORP-AF registry patients eligible for EAST-AFNET 4

*N* (%)	No rhythm control	Early rhythm control	*p*
EAST-AFNET 4 primary outcome	287 (18.5)	245 (13.6)	< 0.001
MACEs	204 (12.5)	176 (9.4)	0.003
All-cause death	191 (11.9)	130 (6.7)	< 0.001

*AF* atrial fibrillation, *CV* cardiovascular, *MACEs* major adverse cardiovascular events

Finally, a Cox regression analysis was performed to establish the association between use of an early rhythm control strategy and the risks of primary and secondary outcomes (Table 5). While the univariate analysis showed that prescription of an early rhythm control strategy was associated with a lower risk for all primary and secondary outcomes, the progressive adjustment process (Model 1 adjusted for type of AF, EHRA score, CHA_2_DS2-VASc score and use of OAC; Model 2 adjusted for type of AF EHRA score, all comorbidities evaluated at baseline and use of OAC) showed a gradual and subsequent loss in association, with the fully adjusted Model 2 showing no significant differences between patients treated with and without an early rhythm control strategy (HR 0.84, 95% CI 0.66–1.19 for EAST-AFNET 4 defined primary outcome; HR 0.95, 95% CI 0.73–1.12 for MACEs; HR 0.96, 95% CI 0.75–1.24 for all-cause death).

**Table 5:** Cox regression analysis for main adverse events for those receiving early rhythm control versus no rhythm control

	Univariate	Model 1*	Model 2^†^
	HR [95% CI]	HR [95% CI]	HR [95% CI]
EAST-AFNET 4 primary outcome	0.69 [0.57–0.83]	0.83 [0.68–1.01]	0.84 [0.66–1.19]
MACEs	0.70 [0.55–0.89]	0.88 [0.69–1.13]	0.95 [0.73–1.24]
All-cause death	0.56 [0.45–0.70]	0.73 [0.58–0.93]	0.96 [0.75–1.24]

*HR* hazard ratio, *MACEs* major adverse cardiovascular events; for other acronyms please see previous tables’ legends

*Adjusted for type of AF, EHRA score, CHA_2_DS2-VASc score, use of OAC

^†^Adjusted for type of AF, EHRA score, age, sex, hypertension, diabetes mellitus, heart failure, severe coronary artery disease, valvular disease, left ventricular hypertrophy, peripheral artery disease, stroke/transient ischaemic attack, chronic kidney disease, chronic obstructive pulmonary disease, malignancy, use of OAC

In the final multivariable model for the EAST-AFNET 4 defined primary outcome, heart failure was associated with a nonsignificant trend for increased risk (HR: 1.21, 95% CI 0.98–1.50, *p* = 0.075). For the occurrence of MACEs, paroxysmal AF was associated with an increased risk of events compared to persistent AF (HR: 1.42, 95% CI 1.02–1.98, *p* = 0.040).

### Sensitivity analysis

We conducted a sensitivity analysis comparing the occurrence of the primary outcome in patients managed with early rhythm control and those with no rhythm control approach but adherent to the ABC pathway management. We selected all patients with data on ABC pathway adherence (2532, 67.1%) and compared those managed with early rhythm control (1356, 53.6%), to those not treated with rhythm control but managed with a clinical management adherent to the ABC pathway (344, 13.6%).

During follow-up, no difference in the cumulative risk of primary outcome between early rhythm control and no rhythm control adherent to ABC pathway was evident (log-rank: 0.099, *p* = 0.753) (Fig. S1). Cox regression analysis confirmed no association between early rhythm control strategy vs. no rhythm control ABC adherent management in univariate (HR: 1.03, 95% CI 0.71–1.49) or multivariable (HR: 0.99, 95% CI 0.65–1.52) analyses.

## Discussion

In this secondary analysis from the ESC-EHRA EORP-AF General Long-Term Registry, only about one-third of European AF patients would be eligible for the original EAST-AFNET 4 study. Second, patients treated with early rhythm control were more likely to be younger, with less comorbidities, were more symptomatic and had a more established form of AF. Third, early rhythm control was associated with better quality of life during follow-up, but with an increased use of health-care resources, as well as a higher risk of hospital readmissions. Fourth, early rhythm control was associated with a lower rate of the composite primary outcome (CV death, any stroke, acute coronary syndrome or worsening heart failure). In the fully adjusted analyses, there were no statistical differences for the primary outcome, MACEs, and all-cause death for patients treated with early rhythm control (Graphical Abstract).

The debate comparing rate vs. rhythm control has been running for the last 20 years. In the ‘Atrial Fibrillation Follow-Up Investigation of Rhythm Management’ study, 4060 AF patients were randomized to rate vs. rhythm control strategies to establish if there were any differences in all-cause death. After 6 years of follow-up, this large trial did not find any differences in mortality between the two groups [22]. In the subsequent years, several other studies have shown a significant reduction of stroke risk and cardiovascular events, in patients managed with rhythm control strategies [23]. Conversely, other studies derived from observational registries report differences in event rates between patients treated with rate or rhythm control strategies, but these differences were no longer significant after adjustment for baseline characteristics [5, 24]. Several large meta-analyses have failed to show any significant differences in clinical outcomes between rate and rhythm control strategies [3, 4].

Other data from the ‘Outcomes Registry for Better Informed Treatment of Atrial Fibrillation’ and EORP-AF General Pilot registries report how in real-world clinical practice, rhythm control strategies are more commonly prescribed to younger and generally healthier patients, perhaps directly influencing the lower rate of events in multivariate analysis [5, 24]. Nonetheless, the older trials were conducted > 2 decades ago and, since then, there has been greater recognition and understanding of electromechanical substrate (and the possibility of early interventions to mitigate the so-called ‘atrial cardiomyopathy’ [25]) as well as the availability of improved (safer) drug therapies and catheter ablation approaches. Indeed, trial data regarding the use of catheter ablation in selected AF patients with heart failure show that using a rhythm control strategy may be associated with a lower rate of major adverse events [26, 27].

On this basis, the EAST-AFNET 4 trial investigators postulated that an early rhythm control could minimize AF-induced atrial damage, hence reducing the occurrence of clinical adverse events [20]. In this trial involving 135 centres, a total of 2789 patients were enrolled and randomized to receive early rhythm control or usual care and followed up for a median of 5.1 years. The early rhythm control arm was associated with a significant reduction in the composite primary outcome of CV death/stroke/hospitalization for heart failure or acute coronary syndrome (HR 0.79, 95% CI 0.66–0.94), particularly driven by reduction in CV death and stroke [7].

In the current analysis of the EORP-AF registry, a large cohort of European AF patients, only one-third of patients could be considered for management with early rhythm control based on the EAST-AFNET 4 criteria. Among those patients qualifying for early rhythm control, such patients were younger and with less comorbidities, consistent with an early rhythm control intervention recommended to younger and healthier patients. While a difference in risk of major adverse outcomes exists when comparing early rhythm control to no rhythm control, this difference may be partly due to the differences in baseline characteristics, as shown in the fully adjusted model. Our analysis also shows that patients managed with early rhythm control are more commonly monitored and undergo further medical treatments, as underlined by the increased use of health-care resources and the higher risk for hospital readmission. Conversely, an early rhythm control strategy was associated with an overall better quality of life, consistent with previous data [3]. Data from the ‘EdoxabaN vs. warfarin in subjectS UndeRgoing cardiovErsion of atrial fibrillation’ trial show how receiving a cardioversion procedure was significantly associated with an improved patients’ quality of life [28].

Indeed, early rhythm control as part of a structured follow-up regimen could partly explain the differences in clinical outcomes seen in the EAST-AFNET 4 trial, where the lower risk for the primary outcome could have been mediated by a better control and treatment of concomitant conditions [29–31]. Indeed, when directly comparing the early rhythm control group with the ABC pathway adherent patients, the absence of any difference in terms of primary outcome occurrence suggests that the beneficial effect of the early rhythm control could be attributed to a more comprehensive strategy of holistic care/management. Furthermore, the open label design in the EAST-AFNET-4 trial could have influenced recurring (and more common) medical checks.

Moreover, the higher incidence rates of the primary outcome both in patients with no rhythm control and early rhythm control, compared to the original study cohort’s event rates, underlines how our real-life cohort is significantly at higher risk for outcomes compared to that seen in the selected population included within a randomized clinical trial. While this poses some limitations to the generalizability of EAST-AFNET 4 trial in real-life patients, it underlines that general AF patients have strong burden of risk related to the complexity of their overall clinical features, questioning whether the ‘one intervention fits all’ application of an early rhythm control approach irrespective of the clinical presentation is appropriate. Also, this given greater justification for integrated or holistic management of AF patients, consistent with the ESC AF guideline-recommended ABC pathway [1].

Indeed, the use of a rhythm control intervention could be prioritized for those patients with a significant burden of symptoms, as part of a comprehensive management of AF patients. The use of rate and rhythm control to reduce the burden of symptoms is one of the pillars of the ABC pathway [1]. Such a structured approach to holistic AF care, including proactive risk evaluation and management, has been shown to be associated with improved clinical outcomes, especially with a reduction in hospitalizations and clinical events [19, 32–34].

### Limitations

This analysis has some limitations. Being a secondary analysis of an observational registry, the original study design was not powered to examine differences in specific subgroups and for specific rhythm strategies. Since a higher burden of symptoms was associated with use of early rhythm control, we could hypothesize that this could represent a bias influencing the use of health-care resources. The inclusion of EHRA score as covariate in the regression analyses regarding the use of health-care resources would minimize such bias.

Despite the use of adjusted analyses, differences in baseline characteristics could have significantly influenced our results, especially with progressive ageing and incident comorbidities [35–38]. Finally, the non-randomized treatment allocation of patients to the groups examined clearly limits the generalizability of our conclusions, raising the need of interpreting our data with caution. Furthermore, while we fully recognize that observational data are no substitute for clinical trials, we analysed a real-world European cohort that is nearly twofold in size compared to the original EAST-AFNET 4 trial, reflecting the generalizability of our results rather than the selective inclusion/exclusion criteria of trial cohorts.

## Conclusions

In this secondary analysis of a large contemporary cohort of AF patients, only one-third of patients would have been eligible for inclusion into the EAST-AFNET 4 trial. Use of an early rhythm control strategy was associated with a lower rate of major adverse events, but this difference was non-significant on multivariate analysis, being mediated by differences in baseline characteristics and clinical risk profile. Early rhythm control was associated with a higher use of health-care resources and risk of hospital admission, despite showing better quality of life.

## Supplementary Information

Below is the link to the electronic supplementary material.

Supplementary file1 (DOCX 1713 kb)
