# Supplementary material for: Real-world applicability and impact of early rhythm control for European patients with atrial fibrillation: a report from the ESC-EHRA EORP-AF Long-Term General Registry
Source: Clin Res Cardiol. 2021 Aug 27;111(1):70–84. doi: 10.1007/s00392-021-01914-y (PMC8766399; doi:10.1007/s00392-021-01914-y)
Supplement: Supplementary file 1 — Supplementary file1 (DOCX 1713 kb) [file 392_2021_1914_MOESM1_ESM.docx]

**Real-World Applicability and Impact of Early Rhythm Control for European Patients with Atrial Fibrillation: A Secondary Analysis from the ESC-EHRA EORP-AF Long-Term General Registry**

Marco Proietti^1,2,3^ MD PhD, Marco Vitolo^1,4^ MD, Stephanie L Harrison^1^ MSc PhD,

Deirdre A Lane^1,5^ PhD, Laurent Fauchier^6^ MD PhD, Francisco Marin^7^ MD PhD, Michael Nabauer^8^ MD, Tatjana S Potpara^9,10^ MD PhD, Gheorghe-Andrei Dan^11^ MD, Giuseppe Boriani^4^† MD PhD, Gregory YH Lip^1,5^† MD

on behalf of the ESC-EHRA EORP-AF Long-Term General Registry Investigators^12^

^1^Liverpool Centre for Cardiovascular Science, University of Liverpool and Liverpool Heart & Chest Hospital, Liverpool, United Kingdom; ^2^Department of Clinical Sciences and Community Health, University of Milan, Milan, Italy; ^3^Geriatric Unit, Fondazione IRCCS Ca’ Granda Ospedale Maggiore Policlinico, Milan, Italy; ^4^Cardiology Division, Department of Biomedical, Metabolic and Neural Sciences, University of Modena and Reggio Emilia, Policlinico di Modena, Modena, Italy; ^5^Aalborg Thrombosis Research Unit, Department of Clinical Medicine, Aalborg University, Aalborg, Denmark; ^6^Service de Cardiologie, Centre Hospitalier Universitaire Trousseau, Tours, France; ^7^Department of Cardiology, Hospital Universitario Virgen de la Arrixaca, IMIB-Arrixaca, University of Murcia, CIBER-CV, Murcia, Spain; ^8^Department of Cardiology, Ludwig-Maximilians-University, Munich, Germany; ^9^School of Medicine, University of Belgrade, Belgrade, Serbia; ^10^Intensive Arrhythmia Care, Cardiology Clinic, Clinical Center of Serbia, Belgrade, Serbia; ^11^'Carol Davila' University of Medicine, Colentina University Hospital, Bucharest, Romania; ^12^See Appendix in Supplementary Materials.

†joint senior authors

Corresponding Author: Prof. Gregory YH Lip

E‐mail: [gregory.lip@liverpool.ac.uk](mailto:gregory.lip@liverpool.ac.uk)

Table S1: Logistic Regression for the Use of Early Rhythm Control

|  | **Univariate** | | **Multivariate** | |
| --- | --- | --- | --- | --- |
|  | OR (95% CI) | p | OR (95% CI) | p |
| **Age** *(per 10 years)* | 0.68 (0.64-0.73) | <0.001 | **0.74 (0.69-0.79)** | **<0.001** |
| **Male Sex** | 1.22 (1.07-1.39) | 0.002 | 1.15 (0.99-1.33) | 0.062 |
| **BMI** *(continuous)* | 1.01 (1.00-1.02) | 0.141 | - | - |
| **Type of AF**  First Detected  Paroxysmal  Persistent | Ref.  1.30 (1.11-1.52)  2.29 (1.93-2.72) | Ref.  0.001  <0.001 | **Ref.**  **1.27 (1.07-1.51)**  **2.20 (1.82-2.65)** | **Ref.**  **0.008**  **<0.001** |
| **EHRA Score** *(continuous)* | 1.40 (1.30-1.52) | <0.001 | **1.49 (1.36-1.63)** | **<0.001** |
| **Hypertension** | 1.24 (1.08-1.42) | 0.002 | **1.36 (1.17-1.59)** | **<0.001** |
| **Diabetes Mellitus** | 0.75 (0.65-0.88) | <0.001 | **0.79 (0.67-0.94)** | **0.008** |
| **NYHA Class** *(continuous)* | 0.89 (0.84-0.94) | <0.001 | **0.88 (0.82-0.93)** | **<0.001** |
| **Stroke/TIA** | 0.81 (0.65-1.00) | 0.050 | **0.78 (0.61-0.99)** | **0.045** |
| **Valvular Disease** | 0.89 (0.78-1.01) | 0.064 | 0.91 (0.78-1.05) | 0.200 |
| **LVH** | 0.87 (0.75-1.01) | 0.071 | 0.88 (0.75-1.03) | 0.112 |
| **CKD** | 0.76 (0.63-0.93) | 0.007 | 0.96 (0.76-1.21) | 0.709 |
| **COPD** | 0.53 (0.41-0.68) | <0.001 | **0.64 (0.49-0.85)** | **0.002** |
| **Any Malignancy** | 0.73 (0.57-0.93) | 0.009 | 0.87 (0.67-1.14) | 0.318 |

Legends: bold values depict significant association; AF= Atrial Fibrillation; BMI= Body Mass Index; CAD= Coronary Artery Disease; CKD= Chronic Kidney Disease; COPD= Chronic Obstructive Pulmonary Disease; EHRA= European Heart Rhythm Association; HF= Heart Failure; LVH= Left Ventricular Hypertrophy; NYHA= New York Heart Association; TIA= Transient Ischemic Attack.

Figure S1: Kaplan-Meier Curves for Primary Outcome Comparing Patients on Early Rhythm Control and in No Rhythm Control ABC Adherent


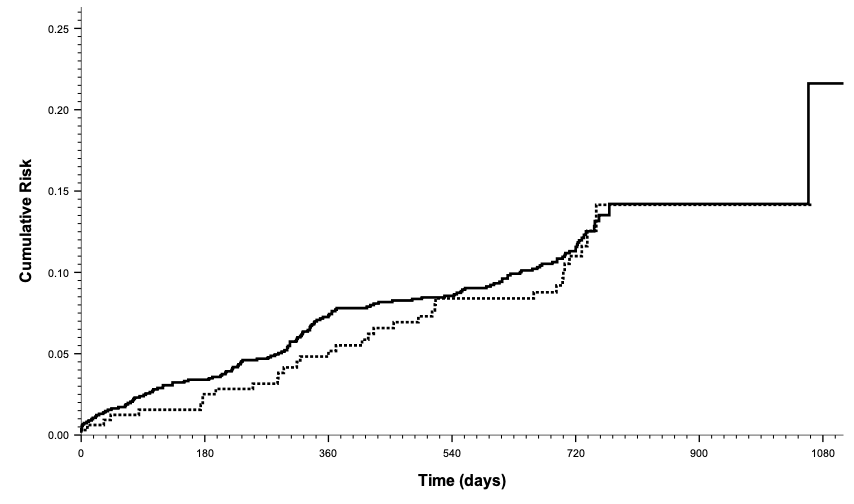


Legend: Black Solid Line= Early Rhythm Control; Black Dotted Line= No Rhythm Control ABC Adherent; ABC= Atrial Fibrillation Better Care.

**APPENDIX**

**EURObservational Research Programme Atrial Fibrillation (EORP-AF) Long-Term General Registry Committees and Investigators**

**Executive committee:** G. Boriani (Chair), G.Y.H. Lip, L. Tavazzi, A. P. Maggioni, G-A. Dan, T. Potpara, M. Nabauer, F. Marin, Z. Kalarus, L. Fauchier, R. Ferrari, A. Shantsila.

**Steering Committee (National Coordinators):** A. Goda, G. Mairesse, T.Shalganov, L. Antoniades, M. Taborsky, S. Riahi, P. Muda, I. García Bolao, O. Piot, M. Nabauer, K. Etsadashvili, E.Simantirakis, M. Haim, A. Azhari, J. Najafian, M. Santini, E. Mirrakhimov, K.a Kulzida, A. Erglis, L. Poposka, M. Burg, H. Crijns, Ö. Erküner, D. Atar, R. Lenarczyk, M. Martins Oliveira, D. Shah, G-A. Dan, E. Serdechnaya, T. Potpara, E. Diker, G.Y.H. Lip, D. Lane.

**Investigators:** **ALBANIA** Durrës: E. Zëra, Tirana: U. Ekmekçiu, V. Paparisto, M. Tase, Tirana: H. Gjergo, J. Dragoti, A. Goda, **BELGIUM** Bastogne: M. Ciutea, N. Ahadi, Z. el Husseini, M. Raepers, Gilly: J. Leroy, P. Haushan, A. Jourdan, Haine Saint Paul: C. Lepiece, Hasselt: L. Desteghe, J. Vijgen, P. Koopman, G. Van Genechten, H. Heidbuchel, Kortrijk: T. Boussy, M. De Coninck, H. Van Eeckhoutte, N. Bouckaert, La Louviere: A. Friart, J. Boreux, C. Arend, Liege: P. Evrard, Liège: L. Stefan, E. Hoffer, J. Herzet, M. Massoz, Liège: C. Celentano, M. Sprynger, L. Pierard, Liège: P. Melon, Overpelt: B. Van Hauwaert, C. Kuppens, D. Faes, D. Van Lier, A. Van Dorpe, Waremme: A. Gerardy, Yvoir: O. Deceuninck, O. Xhaet, F. Dormal, E. Ballant, D. Blommaert, **BULGARIA** Pleven: D. Yakova, M. Hristov, T. Yncheva, N. Stancheva, S. Tisheva, Plovdiv: M. Tokmakova, F. Nikolov, D. Gencheva, Sofia: T. Shalganov, B. Kunev, M. Stoyanov, Sofia: D. Marchov, V. Gelev, V. Traykov, Varna: A. Kisheva, H. Tsvyatkov, R. Shtereva, S. Bakalska-Georgieva, S. Slavcheva, Y. Yotov, **CZECH REPUBLIC** Ústí nad Labem: M. Kubíčková, **DENMARK** Aalborg: A. Marni Joensen, A. Gammelmark, L. Hvilsted Rasmussen, P. Dinesen, S. Riahi, S. Krogh Venø, B. Sorensen, A. Korsgaard, K. Andersen, C. Fragtrup Hellum, Esbjerg: A. Svenningsen, O. Nyvad, P. Wiggers, Herning: O. May, A. Aarup, B. Graversen, L. Jensen, M. Andersen, M. Svejgaard, S. Vester, S. Hansen, V. Lynggaard, Madrid: M. Ciudad, Tallinn: R. Vettus, Tartu: P. Muda, **ESTONIA** Elche, Alicante: A. Maestre, Toledo: S. Castaño, **FRANCE** Abbeville: S. Cheggour, Abbeville: J. Poulard, V. Mouquet, S. Leparrée, Aix-en-Provence: J. Bouet, J. Taieb, Amiens: A. Doucy, H. Duquenne, Angers: A. Furber, J. Dupuis, J. Rautureau, Aurillac: M. Font, P. Damiano, Avignon Cedex: M. Lacrimini, Brest: J. Abalea, S. Boismal, T. Menez, J. Mansourati, Chartres: G. Range, H. Gorka, C. Laure, C. Vassalière, Creteil: N. Elbaz, N. Lellouche, K. Djouadi, Montpellier: F. Roubille, D. Dietz, J. Davy, Nimes: M. Granier, P. Winum, C. Leperchois-Jacquey, Paris: H. Kassim, E. Marijon, J. Le Heuzey, Paris: J. Fedida, C. Maupain, C. Himbert, E. Gandjbakhch, F. Hidden-Lucet, G. Duthoit, N. Badenco, T. Chastre, X. Waintraub, M. Oudihat, J. Lacoste, C. Stephan, Pau: H. Bader, N. Delarche, L. Giry, Pessac: D. Arnaud, C. Lopez, F. Boury, I. Brunello, M. Lefèvre, R. Mingam, M. Haissaguerre, Rennes: M. Le Bidan, D. Pavin, V. Le Moal, C. Leclercq, Saint Denis: O. Piot, T. Beitar, Saint Etienne: I. Martel, A. Schmid, N. Sadki, C. Romeyer-Bouchard, A. Da Costa, Tours: I. Arnault, M. Boyer, C. Piat, L. Fauchier, **FYR MACEDONIA** Bitola: N. Lozance, S. Nastevska, Ohrid: A. Doneva, B. Fortomaroska Milevska, B. Sheshoski, K. Petroska, N. Taneska, N. Bakrecheski, Skopje: K. Lazarovska, S. Jovevska, V. Ristovski, A. Antovski, Skopje: E. Lazarova, I. Kotlar, J. Taleski, L. Poposka, S. Kedev, Skopje: N. Zlatanovik, Štip: S. Jordanova, T. Bajraktarova Proseva, S. Doncovska, **GEORGIA** Tbilisi: D. Maisuradze, A. Esakia, E. Sagirashvili, K. Lartsuliani, N. Natelashvili, N. Gumberidze, R. Gvenetadze, Tbilisi: K. Etsadashvili, N. Gotonelia, N. Kuridze, Tbilisi: G. Papiashvili, I. Menabde, **GERMANY** Aachen: S. Glöggler, A. Napp, C. Lebherz, H. Romero, K. Schmitz, M. Berger, M. Zink, S. Köster, J. Sachse, E. Vonderhagen, G. Soiron, K. Mischke, Bad Reichenhall: R. Reith, M. Schneider, Berlin: W. Rieker, Biberach: D. Boscher, A. Taschareck, A. Beer, Boppard: D. Oster, Brandenburg: O. Ritter, J. Adamczewski, S. Walter, Chemnitz: A. Frommhold, E. Luckner, J. Richter, M. Schellner, S. Landgraf, S. Bartholome, Chemnitz: R. Naumann, J. Schoeler, Dachau: D. Westermeier, F. William, K. Wilhelm, M. Maerkl, Detmold: R. Oekinghaus, M. Denart, M. Kriete, U. Tebbe, Ebersbach: T. Scheibner, Erlangen: M. Gruber, A. Gerlach, C. Beckendorf, L. Anneken, M. Arnold, S. Lengerer, Z. Bal, C. Uecker, H. Förtsch, S. Fechner, V. Mages, Friedberg: E. Martens, H. Methe, Göttingen: T. Schmidt, Hamburg: B. Schaeffer, B. Hoffmann, J. Moser, K. Heitmann, S. Willems, S. Willems, Hartmannsdorf: C. Klaus, I. Lange, Heidelberg: M. Durak, E. Esen, Itzehoe: F. Mibach, H. Mibach, Kassel: A. Utech, Kirchzarten: M. Gabelmann, R. Stumm, V. Ländle, Koblenz: C. Gartner, C. Goerg, N. Kaul, S. Messer, D. Burkhardt, C. Sander, R. Orthen, S. Kaes, Köln: A. Baumer, F. Dodos, Königsbrück: A. Barth, G. Schaeffer, Leisnig: J. Gaertner, J. Winkler, Leverkusen: A. Fahrig, J. Aring, I. Wenzel, Limburg: S. Steiner, A. Kliesch, E. Kratz, K. Winter, P. Schneider, Ludwigsburg: A. Haag, I. Mutscher, R. Bosch, Markkleeberg: J. Taggeselle, S. Meixner, Meissen: A. Schnabel, Meppen: A. Shamalla, H. Hötz, A. Korinth, Merzig: C. Rheinert, Moosburg: G. Mehltretter, Mühldorf: B. Schön, N. Schön, A. Starflinger, E. Englmann, Munich: G. Baytok, T. Laschinger, G. Ritscher, Munich: A. Gerth, Münster: D. Dechering, L. Eckardt, Nienburg: M. Kuhlmann, N. Proskynitopoulos, Paderborn: J. Brunn, K. Foth, Pirna: C. Axthelm, H. Hohensee, K. Eberhard, S. Turbanisch, Plauen: N. Hassler, A. Koestler, Riesa: G. Stenzel, Riesa: D. Kschiwan, M. Schwefer, S. Neiner, S. Hettwer, Rotenburg a.d. Fulda: M. Haeussler-Schuchardt, R. Degenhardt, S. Sennhenn, S. Steiner, Starnberg: M. Brendel, Westerstede: A. Stoehr, W. Widjaja, S. Loehndorf, A. Logemann, J. Hoskamp, J. Grundt, Zorneding: M. Block, Zwiesel: R. Ulrych, A. Reithmeier, V. Panagopoulos, **ITALY** Bologna: C. Martignani, D. Bernucci, E. Fantecchi, I. Diemberger, M. Ziacchi, M. Biffi, P. Cimaglia, J. Frisoni, G. Boriani, Firenze: I. Giannini, S. Boni, S. Fumagalli, S. Pupo, A. Di Chiara, P. Mirone, Modena: E. Fantecchi, G. Boriani, F. Pesce, C. Zoccali, V.L. Malavasi, **KAZAKHSTAN** Almaty: A. Mussagaliyeva, B. Ahyt, Z. Salihova, K. Koshum-Bayeva, **KYRGYZSTAN** Bishkek: A. Kerimkulova, A. Bairamukova, E. Mirrakhimov, **LATVIA** Riga: B. Lurina, R. Zuzans, S. Jegere, I. Mintale, K. Kupics, K. Jubele, A. Erglis, O. Kalejs, **MALTA** Birkirkara: K. Vanhear, M. Burg, M. Cachia, E. Abela, S. Warwicker, T. Tabone, R. Xuereb, **MONTENEGRO** Podgorica: D. Asanovic, D. Drakalovic, M. Vukmirovic, N. Pavlovic, L. Music, N. Bulatovic, A. Boskovic, **NETHERLANDS** Almere: H. Uiterwaal, N. Bijsterveld, Amsterdam: J. De Groot, J. Neefs, N. van den Berg, F. Piersma, A. Wilde, Delfzijl: V. Hagens, Enschede: J. Van Es, J. Van Opstal, B. Van Rennes, H. Verheij, W. Breukers, Heerenveen: G. Tjeerdsma, R. Nijmeijer, D. Wegink, R. Binnema, Hengelo: S. Said, Maastricht: Ö. Erküner, S. Philippens, W. van Doorn, H. Crijns, Rotterdam: T. Szili-Torok, R. Bhagwandien, P. Janse, A. Muskens, s-Hertogenbosch: M. van Eck, R. Gevers, N. van der Ven, Venlo: A. Duygun, B. Rahel, J. Meeder, **NORWAY** Oslo: A. Vold, C. Holst Hansen, I. Engset, D. Atar, **POLAND** Bytom: B. Dyduch-Fejklowicz, E. Koba, M. Cichocka, Cieszyn: A. Sokal, A. Kubicius, E. Pruchniewicz, Gliwice: A. Kowalik-Sztylc, W. Czapla, Katowice: I. Mróz, M. Kozlowski, T. Pawlowski, M. Tendera, Katowice: A. Winiarska-Filipek, A. Fidyk, A. Slowikowski, M. Haberka, M. Lachor-Broda, M. Biedron, Z. Gasior, Kielce: M. Kołodziej, M. Janion, Kielce: I. Gorczyca-Michta, B. Wozakowska-Kaplon, Łódź: M. Stasiak, P. Jakubowski, T. Ciurus, J. Drozdz, Łódź: M. Simiera, P. Zajac, T. Wcislo, P. Zycinski, J. Kasprzak, Nysa: A. Olejnik, E. Harc-Dyl, J. Miarka, M. Pasieka, M. Ziemińska-Łuć, W. Bujak, Opoczno: A. Śliwiński, A. Grech, J. Morka, K. Petrykowska, M. Prasał, Opole: G. Hordyński, P. Feusette, P. Lipski, A. Wester, Radlin: W. Streb, Rzeszów: J. Romanek, P. Woźniak, M. Chlebuś, P. Szafarz, W. Stanik, Szczecin: M. Zakrzewski, J. Kaźmierczak, Szczecin: A. Przybylska, E. Skorek, H. Błaszczyk, M. Stępień, S. Szabowski, W. Krysiak, M. Szymańska, Tarnów: J. Karasiński, J. Blicharz, M. Skura, Warsaw: K. Hałas, L. Michalczyk, Z. Orski, K. Krzyżanowski, A. Skrobowski, Warsaw: L. Zieliński, M. Tomaszewska-Kiecana, M. Dłużniewski, Warsaw: M. Kiliszek, M. Peller, M. Budnik, P. Balsam, G. Opolski, A. Tymińska, K. Ozierański, A. Wancerz, Warsaw: A. Borowiec, E. Majos, R. Dabrowski, H. Szwed, Zabrze: A. Musialik-Lydka, Zabrze: A. Leopold-Jadczyk, E. Jedrzejczyk-Patej, M. Koziel, R. Lenarczyk, M. Mazurek, Z. Kalarus, Zabrze: K. Krzemien-Wolska, P. Starosta, E. Nowalany-Kozielska, Zakopane: A. Orzechowska, M. Szpot, M. Staszel, **PORTUGAL** Almada: S. Almeida, H. Pereira, L. Brandão Alves, R. Miranda, L. Ribeiro, Carnaxide Lisboa: F. Costa, F. Morgado, P. Carmo, P. Galvao Santos, R. Bernardo, P. Adragão, Santarém: G. Ferreira da Silva, M. Peres, M. Alves, M. Leal, Vila Real: A. Cordeiro, P. Magalhães, P. Fontes, S. Leão, Viseu: A. Delgado, A. Costa, B. Marmelo, B. Rodrigues, D. Moreira, J. Santos, L. Santos, **ROMANIA** Arad: A. Terchet, D. Darabantiu, S. Mercea, V. Turcin Halka, A. Pop Moldovan, Brasov: A. Gabor, B. Doka, G. Catanescu, H. Rus, L. Oboroceanu, E. Bobescu, Bucharest: R. Popescu, A. Dan, A. Buzea, I. Daha, G. Dan, I. Neuhoff, Bucharest: M. Baluta, R. Ploesteanu, N. Dumitrache, M. Vintila, Bucharest: A. Daraban, C. Japie, E. Badila, H. Tewelde, M. Hostiuc, S. Frunza, E. Tintea, D. Bartos, Bucharest: A. Ciobanu, I. Popescu, N. Toma, C. Gherghinescu, D. Cretu, N. Patrascu, C. Stoicescu, C. Udroiu, G. Bicescu, V. Vintila, D. Vinereanu, M. Cinteza, R. Rimbas, Iași: M. Grecu, Oradea: A. Cozma, F. Boros, M. Ille, O. Tica, R. Tor, A. Corina, A. Jeewooth, B. Maria, C. Georgiana, C. Natalia, D. Alin, D. Dinu-Andrei, M. Livia, R. Daniela, R. Larisa, S. Umaar, T. Tamara, M. Ioachim Popescu, Târgu Mureș: D. Nistor, I. Sus, O. Coborosanu, Timișoara: N. Alina-Ramona, R. Dan, L. Petrescu, Timișoara: G. Ionescu, I. Popescu, C. Vacarescu, E. Goanta, M. Mangea, A. Ionac, C. Mornos, D. Cozma, S. Pescariu, **RUSSIAN FEDERATION** Arkhangelsk: E. Solodovnicova, I. Soldatova, J. Shutova, L. Tjuleneva, T. Zubova, V. Uskov, Arkhangelsk: D. Obukhov, G. Rusanova, Arkhangelsk: I. Soldatova, N. Isakova, S. Odinsova, T. Arhipova, Arkhangelsk: E. Kazakevich, E. Serdechnaya, O. Zavyalova, Saint-Petersburg: T. Novikova, Saint-Petersburg: I. Riabaia, S. Zhigalov, Saint-Petersburg: E. Drozdova, I. Luchkina, Y. Monogarova, Vladivostok: D. Hegya, L. Rodionova, L. Rodionova, V. Nevzorova, Vladivostok: I. Soldatova, O. Lusanova, **SERBIA** Belgrade: A. Arandjelovic, D. Toncev, M. Milanov, N. Sekularac, Belgrade: M. Zdravkovic, S. Hinic, S. Dimkovic, T. Acimovic, J. Saric, Belgrade: M. Polovina, T. Potpara, B. Vujisic-Tesic, M. Nedeljkovic, Belgrade: M. Zlatar, M. Asanin, Belgrade: V. Vasic, Z. Popovic, Belgrade: D. Djikic, M. Sipic, V. Peric, B. Dejanovic, N. Milosevic, Belgrade: A. Stevanovic, A. Andric, B. Pencic, M. Pavlovic-Kleut, V. Celic, Kragujevac: M. Pavlovic, M. Petrovic, M. Vuleta, N. Petrovic, S. Simovic, Z. Savovic, S. Milanov, G. Davidovic, V. Iric-Cupic, Niška Banja: D. Simonovic, M. Stojanovic, S. Stojanovic, V. Mitic, V. Ilic, D. Petrovic, M. Deljanin Ilic, S. Ilic, V. Stoickov, Pirot: S. Markovic, Šabac:S. Kovacevic. **SPAIN** Alicante: A. García Fernandez, Benalmadena: A. Perez Cabeza, Córdoba: M. Anguita, Granada: L. Tercedor Sanchez, Huarte: E. Mau, J. Loayssa, M. Ayarra, M. Carpintero, Madrid: I. Roldán Rabadan, Murcia: M. Leal, Murcia: M. Gil Ortega, Murcia: A. Tello Montoliu, E. Orenes Piñero, S. Manzano Fernández, F. Marín, A. Romero Aniorte, A. Veliz Martínez, M. Quintana Giner, Pamplona: G. Ballesteros, M. Palacio, O. Alcalde, I. García-Bolao, San Juan de Alicante: V. Bertomeu Gonzalez, Santiago de Compostela: F. Otero-Raviña, J. García Seara, J. Gonzalez Juanatey, **SWITZERLAND** Geneva: N. Dayal, P. Maziarski, P. Gentil-Baron, D. Shah, **TURKEY** Adana: M. Koç, Afyon: E. Onrat, I. E. Dural, Ankara: K. Yilmaz, B. Özin, Ankara: S. Tan Kurklu, Y. Atmaca, Ankara: U. Canpolat, L. Tokgozoglu, Ankara: A. K. Dolu, B. Demirtas, D. Sahin, Ankara: O. Ozcan Celebi, E. Diker, Antalya: G. Gagirci, Bayraklı/Izmir: U.O.Turk, Bursa: H. Ari, Diyarbakır: N. Polat, N. Toprak, Gaziantep: M. Sucu, Görükle-Bursa: O. Akin Serdar, Istanbul: A. Taha Alper, Istanbul: A. Kepez, Istanbul: Y. Yuksel, Kurupelit - Samsun: A. Uzunselvi, S. Yuksel, M. Sahin, Merkez/Düzce: O. Kayapinar, Mersin: T. Ozcan, Sivas: H. Kaya, M. B. Yilmaz, Trabzon: M. Kutlu, Yüreğir-Adana: M. Demir, **UNITED KINGDOM** Barnstaple: C. Gibbs, S. Kaminskiene, M. Bryce, A. Skinner, G. Belcher, J. Hunt, L. Stancombe, B. Holbrook, C. Peters, S. Tettersell, Birmingham: A. Shantsila, D. Lane, K. Senoo, M. Proietti, K. Russell, P. Domingos, S. Hussain, J. Partridge, R. Haynes, S. Bahadur, R. Brown, S. McMahon, G. Y H Lip, Blackburn: J. McDonald, K. Balachandran, R. Singh, S. Garg, H. Desai, K. Davies, W. Goddard, Blackpool: G. Galasko, I. Rahman, Y. Chua, O. Payne, S. Preston, O. Brennan, L. Pedley, C. Whiteside, C. Dickinson, J. Brown, K. Jones, L. Benham, R. Brady, Carlisle: L. Buchanan, A. Ashton, H. Crowther, H. Fairlamb, S. Thornthwaite, C. Relph, A. McSkeane, U. Poultney, N. Kelsall, P. Rice, T. Wilson, Chertsey: M. Wrigley, R. Kaba, T. Patel, E. Young, J. Law, Cramlington: C. Runnett, H. Thomas, H. McKie, J. Fuller, S. Pick, Exeter: A. Sharp, A. Hunt, K. Thorpe, C. Hardman, E. Cusack, L. Adams, M. Hough, S. Keenan, A. Bowring, J. Watts, Great Yarmouth: J. Zaman, K. Goffin, H. Nutt, Harrogate: Y. Beerachee, J. Featherstone, C. Mills, J. Pearson, L. Stephenson, Huddersfield: S. Grant, A. Wilson, C. Hawksworth, I. Alam, M. Robinson, S. Ryan, Macclesfield: R. Egdell, E. Gibson, M. Holland, D. Leonard, Maidstone: B. Mishra, S. Ahmad, H. Randall, J. Hill, L. Reid, M. George, S. McKinley, L. Brockway, W. Milligan, Manchester: J. Sobolewska, J. Muir, L. Tuckis, L. Winstanley, P. Jacob, S. Kaye, L. Morby, Nottingham: A. Jan, T. Sewell, Poole: C. Boos, B. Wadams, C. Cope, P. Jefferey, Portsmouth: N. Andrews, A. Getty, A. Suttling, C. Turner, K. Hudson, R. Austin, S. Howe, Redhill: R. Iqbal, N. Gandhi, K. Brophy, P. Mirza, E. Willard, S. Collins, N. Ndlovu, Rhyl: E. Subkovas, V. Karthikeyan, L. Waggett, A. Wood, A. Bolger, J. Stockport, L. Evans, E. Harman, J. Starling, L. Williams, V. Saul, Salisbury: M. Sinha, L. Bell, S. Tudgay, S. Kemp, J. Brown, L. Frost, Shrewsbury: T. Ingram, A. Loughlin, C. Adams, M. Adams, F. Hurford, C. Owen, C. Miller, D. Donaldson, H. Tivenan, H. Button, South Shields: A. Nasser, O. Jhagra, B. Stidolph, C. Brown, C. Livingstone, M. Duffy, P. Madgwick, Southampton: P. Roberts, E. Greenwood, L. Fletcher, M. Beveridge, S. Earles, Taunton: D. McKenzie, D. Beacock, M. Dayer, M. Seddon, D. Greenwell, F. Luxton, F. Venn, H. Mills, J. Rewbury, K. James, K. Roberts, L. Tonks, Torquay: D. Felmeden, W. Taggu, A. Summerhayes, D. Hughes, J. Sutton, L. Felmeden, Watford: M. Khan, E. Walker, L. Norris, L. O'Donohoe, Weston-super-Mare: A. Mozid, H. Dymond, H. Lloyd-Jones, G. Saunders, D. Simmons, D. Coles, D. Cotterill, S. Beech, S. Kidd, Wolverhampton: B. Wrigley, S. Petkar, A. Smallwood, R. Jones, E. Radford, S. Milgate, S. Metherell, V. Cottam, Yeovil: C. Buckley, A. Broadley, D. Wood, J. Allison, K. Rennie, L. Balian, L. Howard, L. Pippard, S. Board, T. Pitt-Kerby.
